# Editorial: Cephs*In*Action: Towards Future Challenges for Cephalopod Science

**DOI:** 10.3389/fphys.2019.00980

**Published:** 2019-07-25

**Authors:** Lindy Holden-Dye, Giovanna Ponte, A. Louise Allcock, Erica A. G. Vidal, Ryuta Nakajima, Tarla Rai Peterson, Graziano Fiorito

**Affiliations:** ^1^School of Biological Sciences, University of Southampton, Southampton, United Kingdom; ^2^Association for Cephalopod Research ‘CephRes', Naples, Italy; ^3^Department of Biology and Evolution of Marine Organisms, Stazione Zoologica Anton Dohrn, Naples, Italy; ^4^Ryan Institute, National University of Ireland Galway, Galway, Ireland; ^5^Centro de Estudos do Mar, Universidade Federal do Paraná (UFPR), Pontal do Paraná, Brazil; ^6^University of Minnesota Duluth, Duluth, MN, United States; ^7^The University of Texas at El Paso, El Paso, TX, United States

**Keywords:** cephalopods, invertebrates, welfare, outreach, communication, neuroscience, physiology

The collection of papers included in this Research Topic represents the outcome of one of the activities of the COST Action FA1301—“A network for improvement of cephalopod welfare and husbandry in research, aquaculture, and fisheries” (Cephs*In*Action)—that operated for 4 years from 2013 to 2017. The idea of a “Cephs*In*Action” Research Topic entitled “Towards Future Challenges for Cephalopod Science” emerged at one of the last meetings of the COST Action FA1301: Cephs*In*Action and CIAC Meeting “Cephalopod Science from Biology to Welfare^1^” (Hellenic Centre for Marine Research, Heraklion, Crete, Greece, 28–31 March 2017), and from some editorial initiatives discussed at that time. This Research Topic (RT) is just one example of several RTs, and indeed other separate papers, dedicated to cephalopod molluscs (Hanke and Osorio, 2018; Ponte et al., 2018)^2^ that have been hosted by Frontiers over the last few years.

This highlighting of cephalopod science is important as it has much to offer not only the life sciences community, but also more broadly the public perception of science and its understanding and relationship with scientific endeavor. To make this contribution, there are logistical challenges facing the cephalopod research community that need to be overcome. Importantly, given that cephalopod science is a relatively small, globally distributed research community, there is a need for rapid and effective mechanisms for exchange of knowledge and resources, encompassing everything from the sharing of laboratory protocols, videos, tissues and samples, and data-sets to innovation in public engagement. This also presents strategic challenges in convincing globally distributed policy makers and funders of the relevance of cephalopods in scientific advances. There are regulatory aspects too, as cephalopods are the only invertebrates whose use is regulated in Europe in a research context (e.g., Fiorito et al., 2014, 2015; Di Cristina et al., 2015), which increases the need for an integrated oversight and direction in terms of ethics and animal welfare (Ponte et al., 2019). This links to the important recognition that cephalopods are not “simple” laboratory animals and that we need to understand their physiology and behavior by intersecting studies in their natural environment with those in standardized settings such as the lab-bench. Only by a better understanding of the normal range of behaviors of distinct species of cephalopods can their welfare be improved. There is the need for better phylogenetic resolution and for more accurate field data to facilitate this.

This Research Topic also aligns with the interests of the cephalopod community in stimulating public interest in cephalopods and their artistic interpretation. This extends to a broader audience that could include chefs and gourmets^3^, fishers and scientists aiming to develop sustainable food resources. School children's natural fascination with cephalopods (e.g., Sperduti et al., 2012) can excite their interest in scientific discovery and encourage them to engage in conversations about the scientific process and what it means. The importance of such conversations cannot be underestimated in a world in which the public needs to be scientifically literate, as they must be equipped to make important socio-economical and political decisions facing the current condition of the world, whilst being confronted with ‘fake news', ‘alternative truths', and when expert opinion is often derided. There is great potential for innovative schemes for public engagement that springs from the natural wonderment the cephalopods incite.

The last five years have been extremely challenging, but also very innovative for cephalopod science and have continued the outstanding tradition of biological contribution with cephalopod molluscs as key players (e.g., Keynes, 1989; De Sio, 2011; Albertin et al., 2012; Garrett and Rosenthal, 2012; Huffard, 2013; Liscovitch-Brauer et al., 2017; Marini et al., 2017; Sanchez et al., 2018).

This Research Topic includes 13 papers from about 40 authors representing ten different countries, thus overlapping with the original parties that contributed to the COST FA1301. Three papers present original data and 10 others are reviews and perspective articles on various topics, as examples of the interest that humans have for these fascinating marine molluscs. This Research Topic offers a journey that spans cephalopod gastronomy (Mouritsen and Styrbæk) offering a glance at the interest among chefs and gastroscientists to explore these organisms as a counterpoint to other seafood, a look at new protein sources to replace meat from land-animal production, and a test of texture and flavor properties of cuttlefish, squid and octopus and how these provide the ground for a variety of culinary transformations. “CephsInAction: Towards Future Challenges for Cephalopod Science” also offers an “OctopusEye,”: a “refracted spectatorship” perspective and conceptual analysis of the film “The Love Life of the Octopus (Les Amours de la pieuvre) (1965)” (Hayward). But cephalopods are also at the boundaries between “science, art and engineering” (Nakajima et al.). They are among the most enthusiastically visited animals in public aquaria, providing a way of communicating science and conservation (Marchio), and offer various “Critical Challenges Ahead” (O'Brien et al.) as envisioned by three junior “researchers who have recently embarked on careers in cephalopod biology” and that provide their suggestions on a variety of topics spanning from genetics, to welfare, behavior, cognition, and neurobiology.

This volume includes studies on the effects of maternal and embryonic stress on the behavior of offspring (*Sepia officinalis*, O'Brien et al.), presenting evidence for age-related differences in defensive behaviors in the sepiolid *Euprymna* (Seehafer et al.), or the development of swimming abilities of paralarvae of *Doryteuthis opalescens* (Vidal et al.). A preliminary analysis of the expression of protocadherins in *Octopus vulgaris* is also included providing the ground for future analysis of the way these genes may drive neural wiring during development and in the cases of biological and neural plasticity in the adult (Styfhals et al.).

Finally, several reviews are included in this Research Topic, (i) examining parasites that cephalopod host, (ii) raising the possibility of the existence of stem cells in cephalopod brains, (iii) considering possible cases of functional and convergent evolution of neural-systems, when compared with vertebrates, and (iv) overviewing the extraordinary and historically well-studied biological cases of tissue and neural regeneration (Deryckere and Seuntjens; Imperadore and Fiorito; Roumbedakis et al.; Shigeno et al.).

In sum, all contributions reflect a broad, interdisciplinary active and vital scientific community.

## Author Contributions

All authors listed have made a substantial, direct and intellectual contribution to the work, and approved it for publication.

### Conflict of Interest Statement

The authors declare that the research was conducted in the absence of any commercial or financial relationships that could be construed as a potential conflict of interest.

## Footnotes

^1^http://www.cephsinaction.org/activities/meetings/year2016/cephsinactioncretaquarium/

^2^see also: https://www.frontiersin.org/research-topics/10233/vision-in-cephalopods-part-ii; https://www.frontiersin.org/research-topics/9997/cephalopod-research-across-scales---molecules-to-ecosystems

^3^See for example the Atlantic Area Interreg Project “Cephs and Chefs” https://www.cephsandchefs.com/
